# The Microstructure and Properties of Ni-Si-La_2_O_3_ Coatings Deposited on 304 Stainless Steel by Microwave Cladding

**DOI:** 10.3390/ma16062209

**Published:** 2023-03-09

**Authors:** Shashi Prakash Dwivedi, Shubham Sharma, Kanta Prasad Sharma, Abhinav Kumar, Ashish Agrawal, Rajesh Singh, Sayed M. Eldin

**Affiliations:** 1 G.L. Bajaj Institute of Technology & Management, Greater Noida 201310, India; 2 Mechanical Engineering Department, University Centre for Research and Development, Chandigarh University, Mohali 140413, India; 3 School of Mechanical and Automotive Engineering, Qingdao University of Technology, Qingdao 266520, China; 4 Institute of Engineering & Technology, GLA University, Mathura 281406, India; 5 Department of Nuclear and Renewable Energy, Ural Federal University Named after the First President of Russia, Boris Yeltsin, 19 Mira Street, 620002 Ekaterinburg, Russia; 6 Department of Mechanical and Industrial Engineering, Manipal Institute of Technology, Manipal Academy of Higher Education, Manipal, Udupi 576104, India; 7 Uttaranchal Institute of Technology, Uttaranchal University, Dehradun 248007, India; 8 Department of Project Management, Universidad Internacional Iberoamericana, Campeche 24560, Mexico; 9 Center of Research, Faculty of Engineering, Future University in Egypt, New Cairo 11835, Egypt

**Keywords:** wear, hardness, corrosion, La_2_O_3_, cladding process, microwave energy

## Abstract

In this investigation, microwave radiation was used alongside a combination of Ni powder, Si powder, and La_2_O_3_ (Lanthanum oxide) powder to create surface cladding on SS-304 steel. To complete the microwave cladding process, 900 W at 2.45 GHz was used for 120 s. “Response surface methodology (RSM)” was utilized to attain the optimal combination of microwave cladding process parameters. The surface hardness of the cladding samples was taken as a response. The optimal combination of microwave cladding process parameters was found to be Si (wt.%) of 19.28, a skin depth of 4.57 µm, irradiation time of 118 s, and La_2_O_3_ (wt.%) of 11 to achieve a surface hardness of 287.25 HV. Experimental surface hardness at the corresponding microwave-cladding-process parameters was found to be 279 HV. The hardness of SS-304 was improved by about 32.85% at the optimum combination of microwave cladding process parameters. The SEM and optical microscopic images showed the presence of Si, Ni, and La_2_O_3_ particles. SEM images of the “cladding layer and surface” showed the “uniform cladding layer” with “fewer dark pixels” (yielding higher homogeneity). Higher homogeneity reduced the dimensional deviation in the developed cladding surface. XRD of the cladded surface showed the presence of FeNi, Ni_2_Si, FeNi_3_, NiSi_2_, Ni_3_C, NiC, and La_2_O_3_ phases. The “wear rate and coefficient of friction” of the developed cladded surface with 69.72% Ni, 19.28% Si, and 11% La_2_O_3_ particles were found to be 0.00367 mm^3^/m and 0.312, respectively. “Few dark spots” were observed on the “corroded surface”. These “dark spots” displayed “some corrosion (corrosion weight loss 0.49 mg)” in a “3.5 wt.% NaCl environment”.

## 1. Introduction

Steel is a widely used material on Earth. Further, steel offers diverse properties to meet a “wide range of applications”. Steel is used in various applications, such as electrical equipment (3%), domestic appliances (3%), metal products (11%), the automotive industry (12%), mechanical equipment, (15%), buildings and infrastructure (51%), and other forms of transport (5%). Steel is also used in buildings in addition to structural applications for heating ventilation and air conditioning systems, as well as items such as shelving, rails, and stairs [1,2]. SS-304 is nearly one of the most adaptable grades of stainless steel. The principle alloying element SS-304 is chromium other than ferrous. Chromium is present in SS-304 in the oxygen form, which creates a thin layer around the metal. This thin layer produces shielding from the outside environment. “Austenitic stainless steels” are a “non-magnetic material” and have a “BCC (Body-Centered Cubic) structure”. “Austenitic stainless steels” can be “work-hardened” but cannot be hardened by heat treatment. SS-304 is one of the most adaptable grades within the austenitic grades. SS-304 stainless steels are used in heat exchangers; automotive and aerospace components; doors and windows; architectural applications, such as cladding and roofing; fasteners and flange manufacturing; food processing equipment, such as wine making, milk processing, and beer brewing equipment. SS-304 stainless steels are also used in chemical containers, kitchen sinks, consumer durables, etc. [3,4].

The “tensile strength, compressive strength, and fatigue strength” of materials increase using ceramic reinforcement particles, and hardness increases with it. However, sometimes there is a need to significantly increase the “surface hardness of the material” as per the application. In such a situation, surface hardness cannot be significantly increased by the development of traditional composites [5]. In this situation, its surface property is improved by coating hard particles on the material. Today, the surface property of materials is being successfully increased using the microwave cladding technique [6].

Microwave energy applications can be observed in various sectors. However, it is currently being used extensively in food processing. Food is heated without any fuel by microwave energy [7]. Microwave energy applications can be also seen in the welding process over the last few years. The use of microwave energy in the welding process is also a very successful technique. Nowadays, microwave energy is also being used extensively in the surface cladding of materials [8]. The substrate undergoes partial dilution during cladding. Even so, microwave cladding is, at present, widely employed for surface engineering technology [9]. Microwave cladding on stainless steel employing a wide array of carbides, ceramic particles, and Ni powder has improved the material’s surface properties [10]. Microwave cladding is created by subjecting prepared powder to a “household microwave oven” with 2.45 GHz frequency and 900 W outputs for an extended length of time.

Metallic materials such as Ni have outstanding corrosion resistance properties at high temperatures and are used to make alloys, such as stainless steel. Metallic materials’ surface properties play inimitable roles in operating environments [11]. Surface coating with Ni metallic material can improve biocompatibility, oil/water separation, wear resistance, self-cleaning, anti-fouling, and corrosion resistance of stainless steel [12,13]. Further, surface coating is a competent and resource-reduction technique used to understand the metallic materials’ surface functionalization. Additionally, ceramic coating is environmentally friendly and has the merits of “mechanical durability, wear resistance, thermal stability, corrosion resistance, simple preparation and low cost” [14,15]. Ceramic materials are divided into two categories according to their compositions, namely non-oxide ceramics and oxide ceramics. Numerous oxide ceramics forms oxide films on the surface of metallic materials, which are beneficial for functional layers and the protection of metallic materials (for example, titanium alloys, stainless steel, and aluminum). Lanthanum oxide (La_2_O_3_) is rare earth element and an inorganic compound. Lanthanum oxide (La_2_O_3_) exhibits thermostability, possesses outstanding wear resistance properties, and has a wide range of applications in electronic and optics fields [16,17]. Furthermore, the corrosion resistance of La_2_O_3_ promoted its use as coating ceramic particles on steel. Similarly, Si is an extremely attractive coating material for metallic materials such as steel and is used as a corrosion and wear-resistant coating. Corrosion is usually found in steel [18]. Hence, protection against the corrosion of steel is very important for economic harmony and for the sake of the environment. A combination of Ni, La_2_O_3_, and Si coating on steel may prevent corrosion and enhance surface properties because of its good corrosion-resistance behavior [19]. A combination of Ni, La_2_O_3_, and Si provides a barrier on the surface of steel in the form of an “oxide layer” [20,21]. Due to these facts, in this study, a combination of Ni, La_2_O_3_, and Si was used so that the surface property of steel could be increased properly.

According to historical reports, only a small number of studies have employed microwave radiation to activate the mixture of Ni, Si, and La_2_O_3_ powder in the “surface cladding of SS-304”. For the cladding process, three different types of cladding particles were taken in this study, which were of Ni, Si, and La_2_O_3_ powder. However, when cladding with random weight percentages of Ni, Si, and La_2_O_3_ powder was performed, cracks and porosity were observed on the surface during cladding by the microwave technique. To avoid this problem, an attempt was made to find out the optimum microwave parameters using response surface methodology (RSM) so that a defect-free cladding layer could be obtained. The microstructure of the cladding layer, homogeneity, mechanical wear, and XRD behavior of the cladding surface were observed at an optimum combination of microwave parameters to identify the mixture of Ni, Si, and La_2_O_3_ powder cladding on SS-304.

## 2. Materials and Methods

### 2.1. Base Material

The basic metal used in this investigation was SS-304. Standard stainless steel 304 is often used. It has a nickel content of 8% to 10.5% and a chromium content of 18% to 20%. Austenitic stainless steel describes this material. When compared to carbon steel, it is a far less conductive substance both “thermally and electrically”. Nevertheless, SS-304 has a basic magnetic property that is lesser than that of steel. It resists corrosion far better than regular steel. Its malleability means it can be shaped for a broad range of purposes. However, corrosion in pits and crevices can develop in chloride-rich conditions. Above 60 °C, stress corrosion cracking might begin to appear. At temperatures of 870 °C in intermittent duty and 925 °C in continuous service, stainless steel 304 exhibits excellent oxidation resistance [22]. If corrosion resistance in water is a need, however, sustained exposure to temperatures between 425 and 860 °C is not suggested. Table 1 displays the observable properties of SS-304 up to 160 mm in diameter/thickness.

### 2.2. Primary Cladding Particle

**Figure 1:**
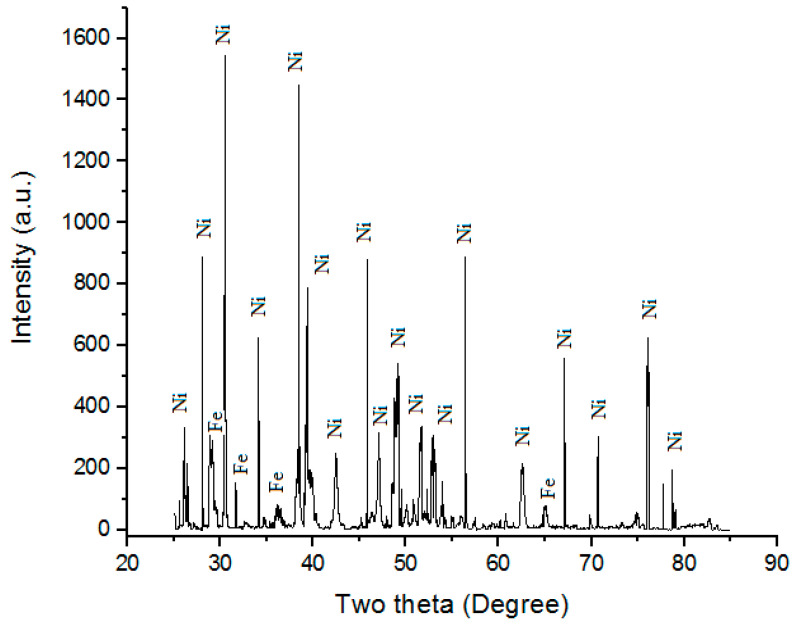
Powder XRD of Ni powder.

Nickel in powdered form, with an “average particle size” of roughly 10 µm, is considered potential “cladding material”. Nickel is a transition metal that is both hard and ductile. In order to optimize the reactive surface area, nickel powder’s high chemical activity is highly beneficial [12]. However, nickel’s ability to passivate steel’s surface after coating it with a protective barrier also makes it resistant to surface corrosion. Because larger Ni pieces take longer to “react with air” under “ordinary circumstances”, an “oxide layer” is not formed on the surface, stopping any further “corrosion”. Only three other elements, “iron, cobalt, and gadolinium”, are magnetic at or near “room temperature”, making it one of the rarest of the rare. Above its “Curie temperature of 355 °C” (671 °F), “bulk nickel is no longer magnetic”. Ni, in comparison to other transition metals, has high thermal and electrical conductivity [23]. In order to determine the purity of the particles, powder XRD was performed on Ni powder used for this investigation. Figure 1 depicts the XRD pattern of Ni powder. When X-ray powder diffraction was performed on Ni powder, almost 99.9% pure Ni was detected. However, Fe phases may also be seen in the Ni powder that was chosen to be the principal cladding particles with SS-304.

### 2.3. Secondary Cladding Particle

**Figure 2:**
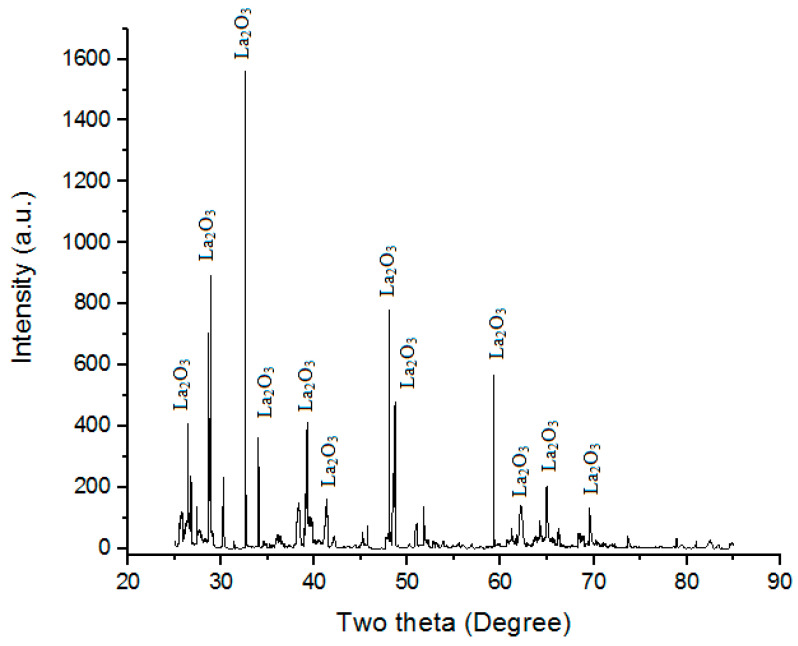
Powder XRD of La_2_O_3_ powder.

Lanthanum oxide (28 µm of particle size) was used along with Ni. It is often used as a component of optical materials in some ferroelectric materials. It is also often used as feedstock for certain catalysts. La_2_O_3_ has an A-M_2_O_3_ hexagonal crystal structure at low temperatures [24]. Powder XRD of La_2_O_3_ particles used in the present study is shown in Figure 2. “Powder XRD” of “La_2_O_3_ powder” shows the “presence of La_2_O_3_ phases”.

### 2.4. Tertiary Cladding Particle

**Figure 3:**
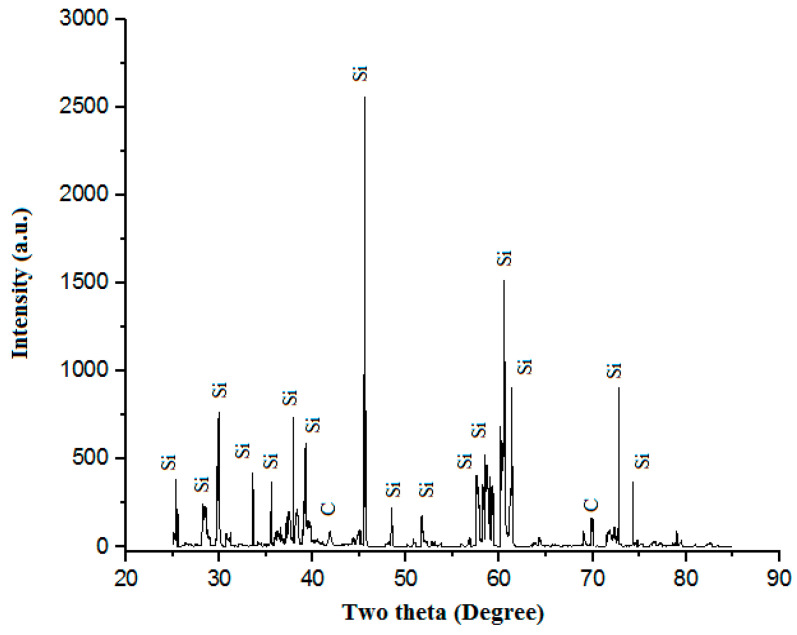
Powder XRD of Si powder.

Silicon powder (Si) was taken as a tertiary cladding particle with an average particle size of 25 µm. It is a semiconductor and a tetravalent metalloid with a blue-grey metallic luster. It is a brittle crystalline that is solid and hard. Its melting temperature is 1414 °C. Silicon powder is inactive at normal temperatures, similar to carbon, but when heated, it reacts dynamically with the halogens (iodine, bromine, chlorine, and fluorine) to form halides and with certain metals to form silicides. Figure 3 shows the “powder XRD” of “Si powder”. “Powder XRD” shows the “presence of Si with a little amount of C”.

### 2.5. Development of Cladding

**Figure 4:**
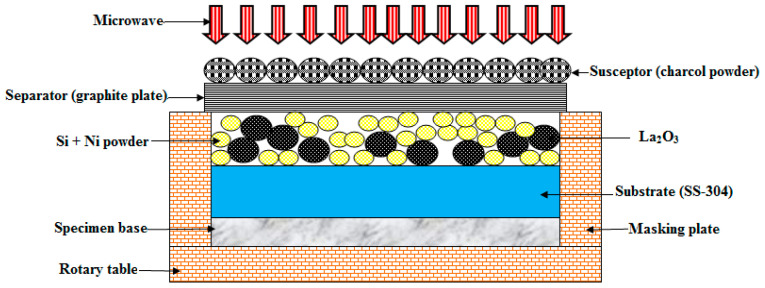
Experimental procedure.

In this study, the “substrate (SS-304) was cleaned” with the help of “alcohol in an ultrasonic bath before deposition”. The mixture of Ni, Si, and La_2_O_3_ particles was preheated at 100 °C for 24 h in a “muffle furnace”. “Preheating of the mixture” of Ni, Si, and La_2_O_3_ particles was carried out to remove the “moisture content of the powder”. The preheated powder was uniformly distributed and preplaced on the “SS-304 substrate” with an “approximately uniform thickness”. However, the “interaction of microwaves” is a highly “material-dependent phenomenon”. In order to overcome the problem of the “microwave being reflected by the mixture of Ni, Si, and La_2_O_3_ particles”, “clads” were developed using “charcoal” as the “susceptor material through microwave hybrid heating (MHH)” [25,26]. The schematic of MHH is shown in Figure 4. MHH was carried out in a “multimode microwave applicator” at 900 W using a “2.45 GHz frequency” [27,28].

### 2.6. Response Surface Methodology (RSM)

RSM was used to identify the optimum combination of microwave cladding process parameters [29,30,31]. In the present study, the central composite design (CCD) in DOE was employed for experimental work [32,33,34]. The weight percent of Si, skin depth, and irradiation time and La_2_O_3_ (wt.%) were obvious based on the “pilot-run investigation”. In the “pilot run”, the arbitrary weight percent of Si was selected as 5% for the microwave cladding process with Ni and La_2_O_3_. It was found that cladding surface hardness was not improved significantly. The weight percent of Si at 15% was found to be satisfactory. However, surface hardness significantly improved by taking 20 wt.% of SiC, along with Ni and La_2_O_3_. Further beyond the Si wt.% of 25, the surface hardness of the cladding layer began to decrease. “Keeping these facts in the mind”, the Si (wt.%) range was kept between 15–25%. Skin depth, irradiation time, and La_2_O_3_ (wt.%) ranges were decided in the same manner. Table 2 shows the “variable process parameters” of “microwave cladding with their ranges”. Table 3 shows the “design matrix table” used to conduct the experiment for surface hardness as response. Standard order (Table 3) shows the non-random sequence of the experimental runs for the fractional factorial design of the experiment in the present study.

### 2.7. Materials Testing Procedure

Microstructures of the clad samples were identified by taking an SEM image. Carbon tape was fixed on two sides of the clad samples, and the UHMWPE powder was sprinkled onto the surface. A light platinum, gold, or carbon coating was used (≈ 100 Å), and the clad samples were examined in an SEM chamber. A small diamond pyramid was used indenter, loaded with a small force of 100 gf for the Vickers hardness test of the clad samples [35,36]. X-ray diffraction (XRD) is the nondestructive instrument used to examine different types of substances, ranging from crystals to powders and fluids. Atoms scatter X-ray waves, first and foremost via the atoms’ electrons. XRD is used to observe the percentage of crystallinity, distinguish between crystalline and amorphous materials, detect a variety of polymorphic forms, and recognize crystalline material. A corrosion test was performed in the presence of 3.5 wt. percent NaCl over 120 h.

## 3. Results and Discussion

### 3.1. Analysis of Variance for the Surface Hardness as a Response

Table 4 shows the ANOVA table used to obtain the “optimal combination of microwave cladding process parameters” for “surface hardness as a response”. In this case, Si (wt.%), skin depth, irradiation time, La_2_O_3_ (wt.%), Si (wt.%)^2^, skin depth^2^, irradiation time^2^, La_2_O_3_ (wt.%)^2^, interaction of Si (wt.%) and skin depth, interaction of Si (wt.%) and irradiation time, interaction of Si (wt.%) and La_2_O_3_ (wt.%), interaction of skin depth and irradiation time, interaction of skin depth and La_2_O_3_(wt.%), and interaction of irradiation time and La_2_O_3_ (wt.%) were significant model terms. The present result showed a ratio of 43.646. This model can be utilized to “navigate the design space”. Equation (1) shows the final equation in terms of actual factors.

Hardness (HV) = −84.39 + 9.73 × Si + 57.96 × Skin depth + 1.95 × Irradiation time + 4.05 × La_2_O_3_ − 0.16 × Si^2^ − 3.04 × Skin depth^2^ − 3.38 × 10^−3^ × Irradiation time^2^ − 0.06 × La_2_O_3_^2^ − 0.28 × Si × Skin depth − 0.01 × Si × Irradiation time − 0.03 × Si × La_2_O_3_ − 0.18 × Skin depth × Irradiation time − 0.38 × Skin depth × La_2_O_3_ − 5.83 × 10^−3^ × Irradiation time × La_2_O_3_ (1)

**Figure 5:**
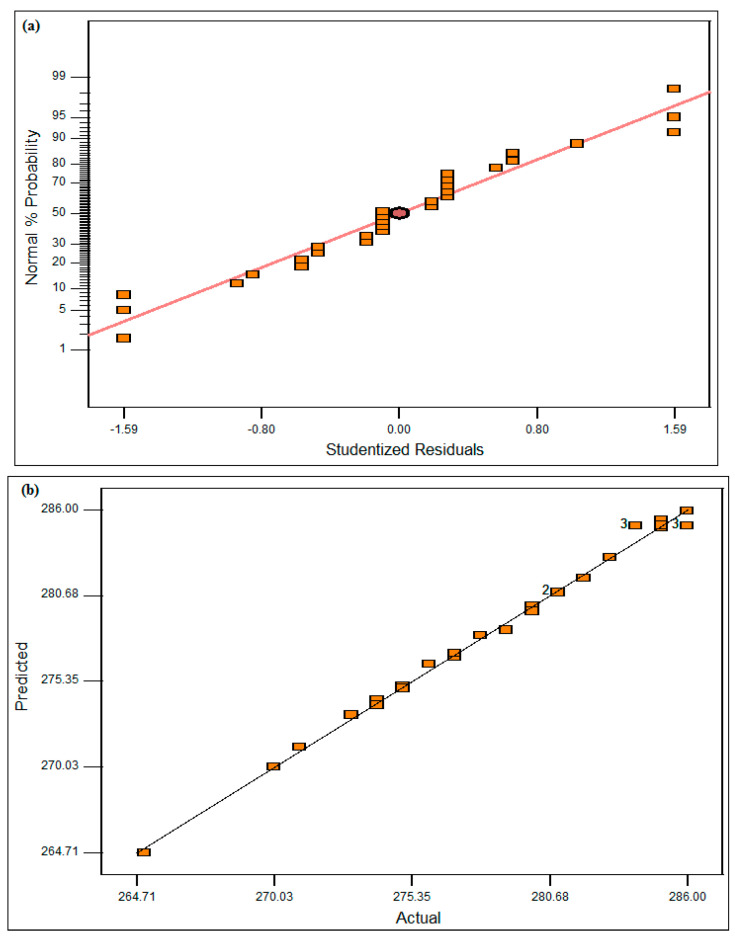
(**a**) Studentized residuals graph; (**b**) actual vs. predicted graph.

The studentized residuals graph and the experimental predicted graph can be seen in Figure 5a,b, respectively. Through these graphs, it is known that the experiment that was conducted is correct through the design matrix table. The present study results show that both graphs appear to be plotted in a straight line of about 45 degrees.

### 3.2. Process Parameters Effect on Cladding Surface Hardness 

#### 3.2.1. Influence of “Silicon Powder Weight” Percent on Cladding Surface Hardness

**Figure 6:**
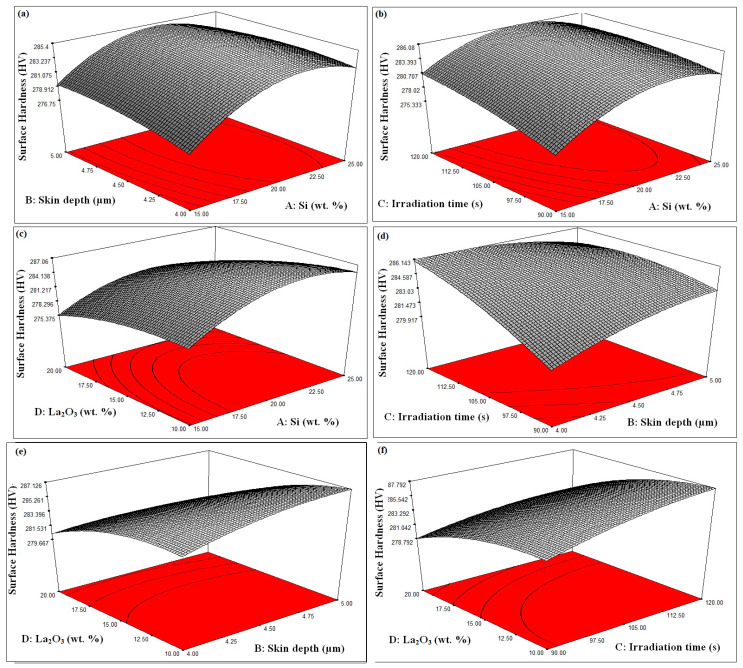
(**a**–**f**). Process Parameters Effect on Surface Hardness.

The influence of “silicon powder weight percentage” on “surface hardness” can be seen in Figure 6a–c. A steady increase in surface hardness was observed when the weight percent of Si powder was taken up to the middle of the selected range. However, when the weight precept was further increased, surface hardness began to decrease. The reason for this may be that the increase in the weight percentage of Si caused some defects to arise during the “formation of the cladding layer”, due to which a “decrease in surface hardness” was observed [36,37,38].

#### 3.2.2. Effect of Skin Depth of the Major Constituent on Cladding Surface Hardness

The effect of skin depth of the major constituent on surface hardness can be observed in Figure 6a,d,e. The value of surface hardness came out best when the “skin depth of the major constituent” was placed approximately between the selected process parameters (4 µm to 5 µm). Having a higher “skin depth of the major constituent” increases the chances of cracking of the substrate during the “cladding process”, resulting in a decrease in the “hardness of the surface” [39,40,41]. Therefore, during the cladding process, it is kept in mind that the “skin depth of the major constituent” should neither be too high nor too low. Keeping the “skin depth of the major constituent” low, sometimes proper cladding does not occur, due to which the required increase in surface hardness is not achieved.

#### 3.2.3. Effect of Irradiation Time on Cladding Surface Hardness

The influence of irradiation time on “surface hardness” is displayed in Figure 6b,d,f. It is visible from Figure 6b,d,f that surface hardness also increases by increasing irradiation time. Microwave irradiation gives more time for cladding particles to bond with the surface, due to which the cladding surface is properly formed [42,43,44].

#### 3.2.4. Effect of La_2_O_3_ Powder Weight Percent on Cladding Surface Hardness

It can be seen from Figure 6c,e,f that surface hardness continuously increases when the weight percentage of La_2_O_3_ increases. The reason for this is that La_2_O_3_ itself is a very hard particle and when its weight percent increases, it tries to increase the hardness of the substrate very well [45,46,47].

### 3.3. Microwave Cladding Optimum Parameters and Contribution

**Figure 7:**
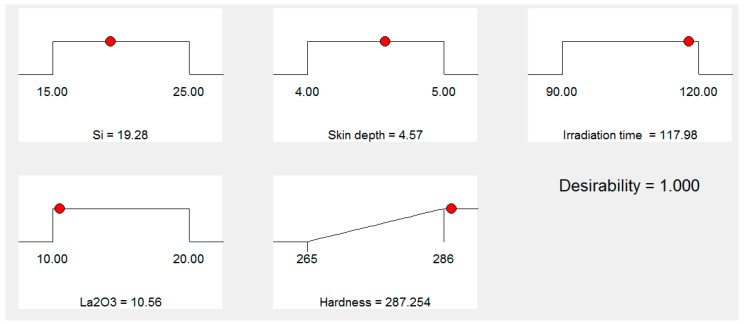
Ramp function graph for the input parameters Si (wt.%), skin depth, irradiation time, La_2_O_3_ (wt.%).

**Figure 8:**
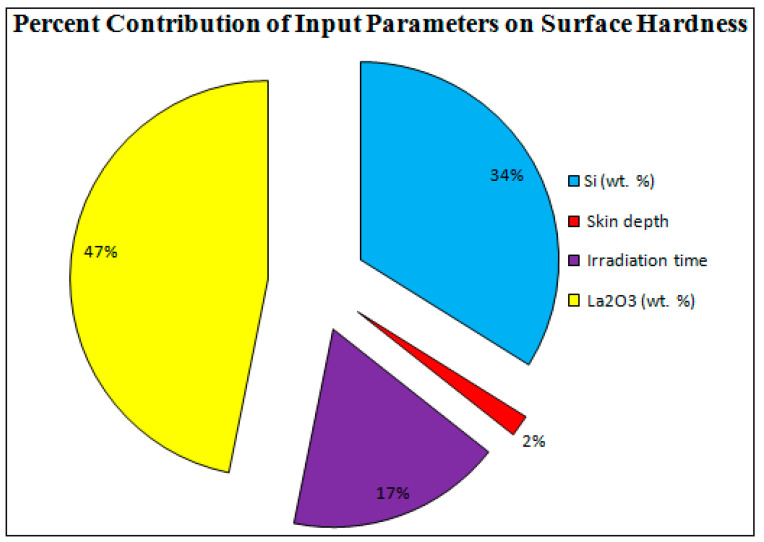
Percent Contribution of Input Parameters.

Figure 7 shows the “ramp function graph” for the “microwave cladding input parameters”. The “ramp function graph results” exhibit that if the values of Si (wt.%), skin depth of the major constituent, irradiation time, and La_2_O_3_ (wt.%) are about 19.28, 4.57 µm, 118 s, and 11, respectively, then the value of cladding surface hardness should be 287.25 HV with a desirability of 1. The prominence of the “microwave cladding parameters” can be ranked based on their F ratio as exhibited in Table 5 (ANOVA table). It can be inferred that La_2_O_3_ (wt.%) contributes the most, followed by Si (wt.%), irradiation time, and skin depth of the major constituent, as shown in Figure 8.

### 3.4. Metallurgical and Tribo-Corrosion Behaviour at Optimum Microwave Process Parameters

**Figure 9:**
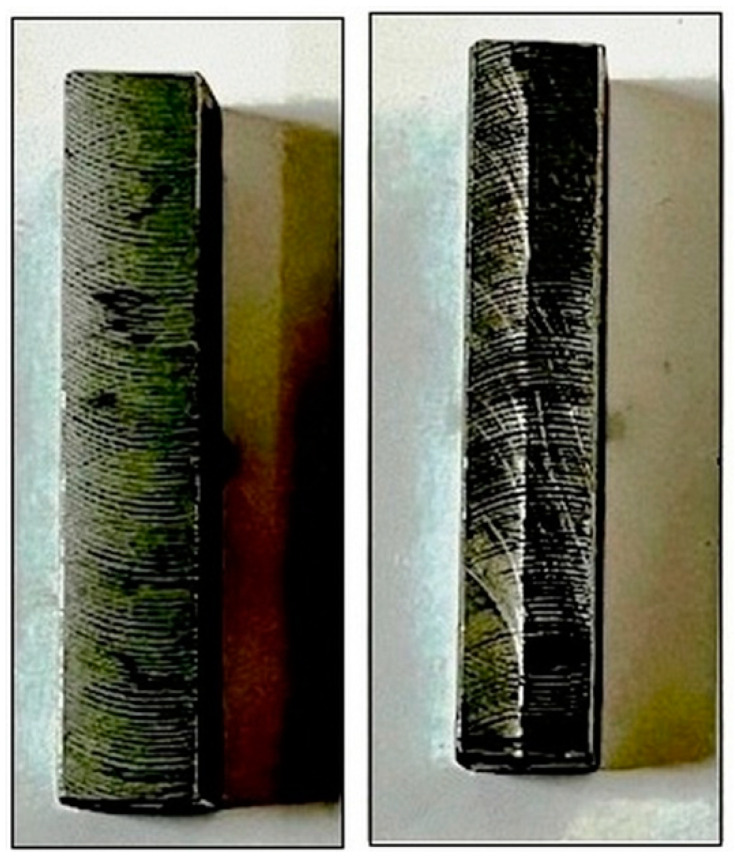
Photograph of the microwave clad samples.

Microwave cladding of SS-304 was carried out at optimum microwave cladding process parameters (Si (wt.%) of 19.28, skin depth of 4.57 µm, and irradiation time of 118 s and La_2_O_3_ (wt.%) of 11). Figure 9 shows the photograph of the “microwave clad samples” developed at “optimum microwave process parameters”. Surface hardness, microstructure investigation, XRD behavior, wear behavior, and corrosion behavior of developed samples at optimum microwave process parameters (Si (wt.%) of 19.28, skin depth of 4.57 µm, irradiation time of 118 s, and La_2_O_3_ (wt.%) of 11) have been discussed as shown below.

#### 3.4.1. Surface Hardness

**Figure 10:**
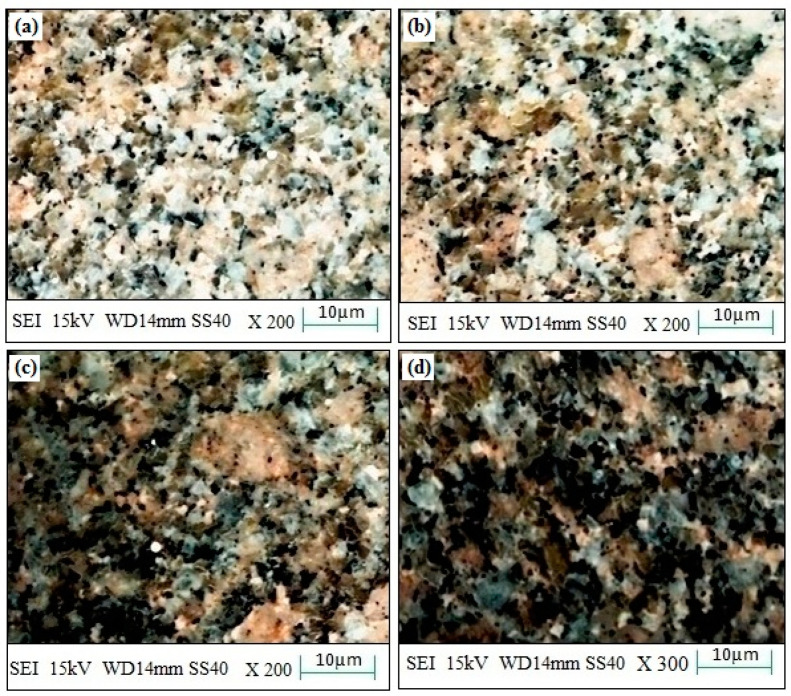
(**a**–**d**). SEM images of the “microwave clad samples” with mixture of 69.72% Ni, 19.28% Si, and 11% La_2_O_3_ particles on “SS-304 developed at optimum cladding parameters”.

**Figure 11:**
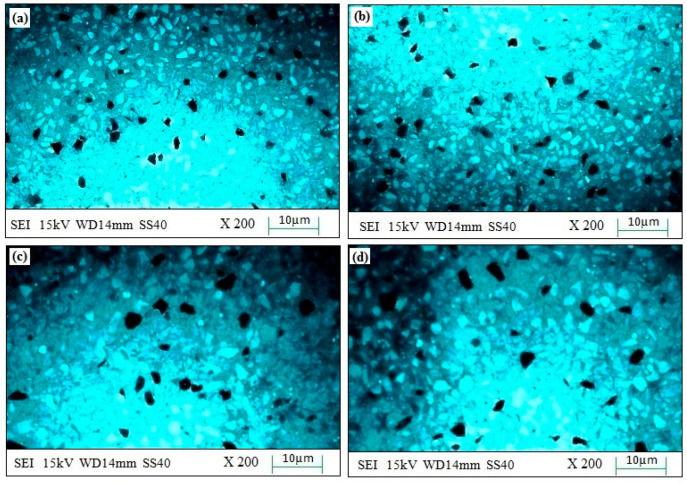
(**a**–**d**). SEM images of the “cladding layer surface” developed using a mixture of 69.72% Ni, 19.28% Si, and 11% La_2_O_3_ particles on “SS-304 developed at optimum cladding parameters”.

**Figure 12:**
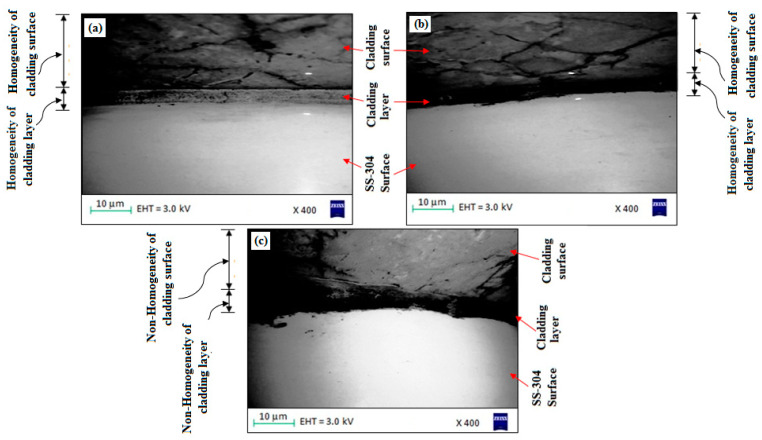
(**a**–**c**). SEM image of “cladding layer and cladding surface” of the “microwave clad samples” developed using a “mixture of 69.72% Ni, 19.28% Si, and 11% La_2_O_3_ particles” on “SS-304 developed at optimum cladding parameters”.

The experimental surface hardness (average for five test samples) corresponding to the microwave cladding process parameters (Si (wt.%) of 19.28, skin depth of 4.57 µm, Irradiation time of 118 s, and La_2_O_3_ (wt.%) of 11) was found to be 279 HV. The ramp function graph shows that the theoretical value of surface hardness at the optimum microwave cladding process parameters is 287.25 HV. The results indicate that there is only a 2.96% error in the experimental and mathematical model results. The average hardness of the SS-304 alloy was observed to be 210HV. The outcomes exhibit that there is about a 32.85% enhancement in the “hardness of SS-304 alloy” after the “microwave cladding of the mixture” of 69.72% Ni, 19.28% Si, and 11% La_2_O_3_ particles on “SS-304 at optimum process parameters” [29,30,31]. In Figure 10a–d, the SEM image displays the "microwave clad samples" produced using a "mixture of 69.72% Ni, 19.28% Si, and 11% La2O3 particles" on "SS-304" that was developed under optimal cladding parameters. The surface of the "SS-304" exhibits a uniform distribution of Ni, Si, and La2O3 particles. Additionally, the presence of La2O3 powder is visible on the steel surface. As demonstrated in Figure 11a–d, the use of Ni and Si powder as a coating material is effective in enhancing the surface properties of steel. The numerous “hard and carbide phases” developed after “microwave cladding”, such as the Ni_3_C, NiC, and La_2_O_3_ phases (Figure 12a–c), were accountable for ornamental hardness [48,49,50].

#### 3.4.2. Microstructure Investigation

Figure 10a–d shows the SEM image of the “microwave clad samples” developed using the “mixture of 69.72% Ni, 19.28% Si, and 11% La_2_O_3_ particles” on “SS-304 developed at optimum cladding parameters”. “Uniform distribution of Ni, Si, and La_2_O_3_ particles” can be observed on the “surface of SS-304”. The presence of La_2_O_3_ powder can be observed on the surface of the steel. “Ni and Si powder” itself is a “good coating material” that enhances the “surface property of steel” (Figure 11a–d). Figure 12a–c shows the “SEM image of the cladding layer and cladding surface”. Figure 12a,b show the “uniform cladding layer” with “fewer dark pixels” (yielding higher homogeneity). The uniform cladding layer and cladding surface were obtained by maintaining the optimum microwave parameters (Si (wt.%) of 19.28, skin depth of 4.57 µm, and Irradiation time of 118 s, and La_2_O_3_ (wt.%) of 11). By retaining the best microwave settings, a uniform cladding layer and surface were produced. The created cladding surface’s dimensional deviation was minimized with increased “cladding layer and cladding surface homogeneity” [30,31]. The hardness of the cladding surface is better distributed when there is less variation in its dimensions. The wear resistance and average hardness of the produced surface are both improved by uniform hardness distribution over the cladding surface. Some fissures between the cladding layer and substrate are seen in Figure 12c. Inconsistent variations in powder and microwave settings may have led to the development of the fracture [51,52,53]. Because of fissures that formed amid the “cladding layer and the substrate”, wear resistance was reduced. On the other hand, formed fissures between the cladding layer and substrate may also reduce hardness [54,55,56].

#### 3.4.3. XRD Behavior

**Figure 13:**
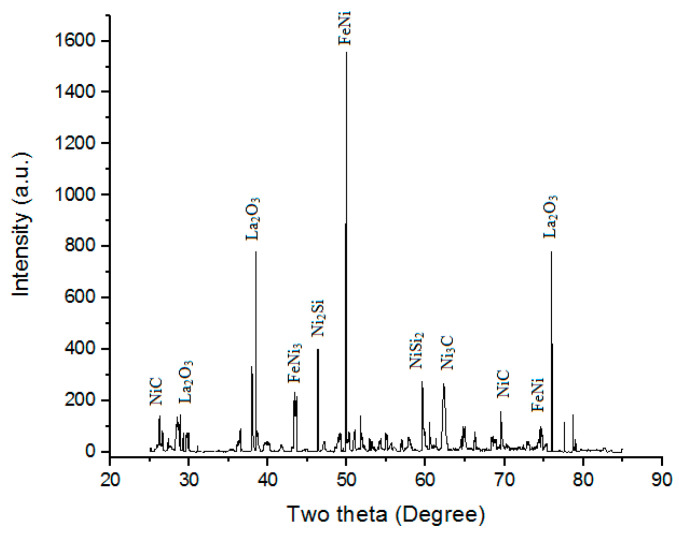
XRD of the “microwave clad samples” developed using a “mixture of 69.72% Ni, 19.28% Si, and 11% La_2_O_3_ particles” on “SS-304 developed at optimum cladding parameters”.

XRD is a prevailing nondestructive method intended to distinguish crystalline materials. It gives information of crystal defects, strain, crystallinity, average grain size, texture, phases, and structures [57,58,59]. Figure 13 shows the XRD of the “microwave clad samples” developed using a “mixture of 69.72% Ni, 19.28% Si, and 11% La_2_O_3_ particles” on “SS-304 developed at optimum cladding parameters”. XRD of cladded surface shows the presence of FeNi, Ni_2_Si, FeNi_3_, NiSi_2_, Ni_3_C, NiC, and La_2_O_3_ phases. The “formation of hard and carbide phases”, such as the Ni_3_C, NiC, and La_2_O_3_ phases, were accountable for ornamental “hardness of SS-304 alloy” after the “cladding of the mixture of 69.72% Ni, 19.28% Si, and 11% La_2_O_3_ particles” [32,33,34].

#### 3.4.4. Wear Behavior

For the wear test, a “pin-on-disc machine” with a “sliding speed of 2 m/s, a sliding distance of 1000 m, and an axial load of 5 N” was used. The “wear rate and coefficient of friction” of the developed “cladded surface” with “69.72% Ni, 19.28% Si, and 11% La_2_O_3_ particles” were observed to be 0.00367 mm^3^/m and 0.312, respectively, which is appropriate to be utilized anywhere. Nevertheless, the foremost purpose for “good wear resistance” is the “use of La_2_O_3_ with Ni and Si powder” in the development of the “cladding surface” [60,61,62]. The development of “hard phases such as Ni_3_C, NiC, and La_2_O_3_ phases” (Figure 12) on the “surface of SS-304” with “microwave cladding of the mixture of 69.72% Ni, 19.28% Si, and 11% La_2_O_3_ particles” was responsible for increased “wear resistance”. However, “La_2_O_3_ is a hard particle” whose presence always promotes “wear resistance of the material”. Wear behavior of the cladding surface of steel depends on many factors, such as cladding thickness, adhesion of cladding to the substrate, layers form after cladding, microstructure (number and size of defects, phases, and grain sizes), thermal properties of cladding and substrate, mechanical properties of cladding and substrate (fatigue strength, tensile strength, Young’s modulus, and hardness), and surface roughness.

#### 3.4.5. Corrosion Behavior

**Figure 14:**
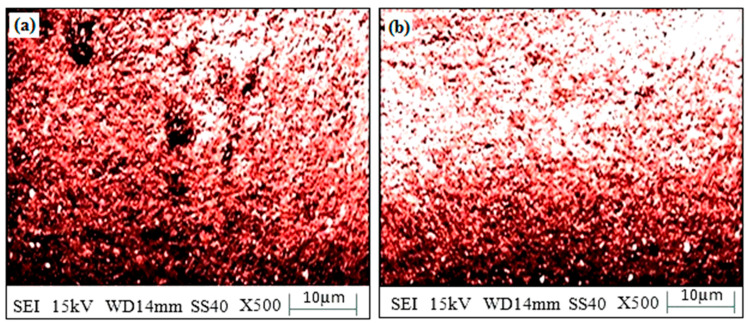
(**a**,**b**). Surface morphology of corroded clad sample developed using a mixture of 69.72% Ni, 19.28% Si, and 11% La_2_O_3_ particles on SS-304 developed at optimum cladding parameters.

In the “existence of 3.5 wt. percent NaCl” over 120 h, the “corrosion test of SS-304” with “microwave cladding” of a “mixture of 69.72% Ni, 19.28% Si, and 11% La_2_O_3_ particles” was conducted. “Corrosion weight loss of SS-304” with “microwave cladding” of a “mixture of 69.72% Ni, 19.28% Si, and 11% La_2_O_3_ particles” was found to be 0.49 mg. Figure 14a,b shows the “SEM image of the corroded surface”. “Few dark spots” may be observed on the “corroded surface”. These “dark spots” indicate some “corrosion in 3.5 wt.% NaCl environment”.

## 4. Conclusions

The subsequent points can be concluded from the current work:

1. The “mixture of 69.72% Ni, 19.28% Si, and 11% La_2_O_3_ particles” can be utilized on “SS-304 alloy for microwave cladding via microwave energy”.
2. RSM results show that if the values of Si (wt.%), skin depth of the major constituent, irradiation time, and La_2_O_3_ (wt.%) are about 19.28, 4.57 µm, 118 s, and 11, respectively, then the value of cladding surface hardness should be 287.25 HV with a desirability of 1. The ANOVA table shows that La_2_O_3_ (wt.%) contributes the most, followed by Si (wt.%), irradiation time, and skin depth of the major constituent.
3. An SEM image of the “microwave clad samples” developed using a “mixture of 69.72% Ni, 19.28% Si and 11% La_2_O_3_ particles” on “SS-304 developed at optimum cladding parameters” showed “uniform distribution of Ni, Si, and La_2_O_3_ particles”. A “Uniform cladding-layer” with “fewer dark pixels” was observed in between the “substrate and cladding surface”.
4. XRD of the cladded surface shows the presence of FeNi, Ni_2_Si, FeNi_3_, NiSi_2_, Ni_3_C, NiC, and La_2_O_3_ phases.
5. The “hardness of the cladding surface” was enhanced by about 32.85%. The foremost purpose for the enhancement in “hardness is the various hard phases”, developed after “microwave cladding such as Ni_3_C, NiC, and La_2_O_3_ phases”.
6. The “wear rate and coefficient of friction” of developed “cladded surfaces” with a mixture of 69.72% Ni, 19.28% Si, and 11% La_2_O_3_ particles on SS-304 developed at optimum cladding parameters were found to be 0.00367 mm^3^/m and 0.312, respectively.
7. “Few dark spots” were observed on the “corroded surface of SS-304” with the “microwave cladding of the mixture of 69.72% Ni, 19.28% Si, and 11% La_2_O_3_ particles”. These “dark spots” displayed some “corrosion in 3.5 wt.% NaCl environment”.
8. Steel plate coating with 69.72% Ni, 19.28% Si, and 11% La_2_O_3_ may be used in the application of condensers for power plants; central air conditioning; and tube sheets used for heat exchangers, reactor columns, and pressure vessels in the oil and gas chemical industries, such as those used in storage tanks, etc.

## Figures and Tables

**Table 1:** Properties of SS-304.

S. No.	Properties	Values
1	“Density (g/cm^3^)”	8
2	“Tensile Strength (MPa)”	615
3	“Melting Temperature (Degree centigrade)”	1450
4	“Vickers hardness (HV)”	210

**Table 2:** Variable process parameters with their ranges.

S. No.	Input Parameters	Range
1	Si (wt.%)	15–25
2	The skin depth (µm)	4–5
3	Irradiation time (s)	90–120
4	La_2_O_3_ (wt.%)	10–20

**Table 3:** Design Matrix Table.

Standard Order	Run	A: Si (wt.%)	B: The Skin Depth of the Major Constituent of the Hard-Facing Powder (µm)	C: Irradiation Time (s)	D: La_2_O_3_ (wt.%)	Surface Hardness (HV)
14	1	25	4	120	20	280
10	2	25	4	90	20	276
18	3	30	4.5	105	15	274
4	4	25	5	90	10	285
2	5	25	4	90	10	281
25	6	20	4.5	105	15	286
13	7	15	4	120	20	277
28	8	20	4.5	105	15	286
21	9	20	4.5	75	15	279
19	10	20	3.5	105	15	281
17	11	10	4.5	105	15	265
29	12	20	4.5	105	15	284
23	13	20	4.5	105	5	285
16	14	25	5	120	20	275
22	15	20	4.5	135	15	285
26	16	20	4.5	105	15	284
12	17	25	5	90	20	277
27	18	20	4.5	105	15	284
3	19	15	5	90	10	278
8	20	25	5	120	10	285
6	21	25	4	120	10	286
24	22	20	4.5	105	25	274
20	23	20	5.5	105	15	283
9	24	15	4	90	20	270
5	25	15	4	120	10	280
11	26	15	5	90	20	273
7	27	15	5	120	10	282
1	28	15	4	90	10	271
15	29	15	5	120	20	275
30	30	20	4.5	105	15	286

**Table 4:** ANOVA Table.

“Source”	“Sum of Squares”	“DF”	“Mean Square”	“F Value”	“Prob > F”	
Model	910.2833333	14	65.0202381	137.689916	<0.0001	significant
A	135.375	1	135.375	286.6764706	<0.0001	
B	7.041666667	1	7.041666667	14.91176471	0.0015	
C	70.04166667	1	70.04166667	148.3235294	<0.0001	
D	187.0416667	1	187.0416667	396.0882353	<0.0001	
A2	414.0744048	1	414.0744048	876.8634454	<0.0001	
B2	15.86011905	1	15.86011905	33.58613445	<0.0001	
C2	15.86011905	1	15.86011905	33.58613445	<0.0001	
D2	52.64583333	1	52.64583333	111.4852941	<0.0001	
AB	7.5625	1	7.5625	16.01470588	0.0012	
AC	14.0625	1	14.0625	29.77941176	<0.0001	
AD	10.5625	1	10.5625	22.36764706	0.0003	
BC	27.5625	1	27.5625	58.36764706	<0.0001	
BD	14.0625	1	14.0625	29.77941176	<0.0001	
CD	3.0625	1	3.0625	6.485294118	0.0223	
“Residual”	7.083333333	15	0.472222222			
“Lack of Fit”	1.083333333	10	0.108333333	0.090277778	0.9992	not significant
“Pure Error”	6	5	1.2			
“Cor Total”	917.3666667	29				
“Std. Dev.”	0.687184271		“R-Squared”	0.992278624		
“Mean”	279.5666667		“Adj R-Squared”	0.985072006		
“C.V.”	0.245803364		“Pred R-Squared”	0.983779659		
“PRESS”	14.88		“Adeq Precision”	43.64638992		

**Table 5:** Wear and friction behavior of SS-304 with and without cladding of the mixture of 69.72% Ni, 19.28% Si, and 11% La_2_O_3_ particles at a sliding speed of 2 m/s and a sliding distance of 1000 m.

Axial Load	Wear Rate (mm^3^/m)		Friction	
	SS-304	The Mixture of 69.72% Ni, 19.28% Si, and 11% La_2_O_3_ Particles Cladding on SS-304	SS-304	The Mixture of 69.72% Ni, 19.28% Si, and 11% La_2_O_3_ Particles Cladding on SS-304
2.5	0.00411	0.00292	0.091	0.294
5	0.00456	0.00367	0.0956	0.312
7.5	0.00471	0.00397	0.0987	0.345
10	0.00490	0.00401	0.0994	0.398

## Data Availability

No data were used to support this study.
